# Cascading model of causal relationships in EMS ontogeny: integration of Piaget, Erikson, Bowlby, and Young paradigms

**DOI:** 10.3389/fpsyt.2026.1880786

**Published:** 2026-08-19

**Authors:** Katarzyna Gutowska-Maślanka, Monika Talarowska-Dublicka

**Affiliations:** Institute of Psychology, Faculty of Educational Sciences, University of Lodz, Lodz, Poland

**Keywords:** biological lock, cascade model, early maladaptive schemas, personality, schema therapy

## Abstract

The article presents a proposal for a model explaining the ontogeny of early maladaptive schemas (EMS) by linking classical developmental theories (Piaget, Erikson, Bowlby) with contemporary findings in the field of epigenetics. The proposed model indicates that EMS development is sequential in nature, involving interrelated cognitive, relational and biological levels. Early relational experiences, especially during periods of increased neurobiological plasticity, lead to permanent changes in the functioning of the hypothalamic–pituitary–adrenal axis and changes in gene expression associated with the regulation of stress responses (e.g., NR3C1, FKBP5). The dominance of assimilation processes over accommodation processes is conducive to the consolidation of rigid cognitive-emotional structures, which, along with the development of identity, take the form of integrated belief systems. The article introduces the concept of “biological lock”, describing the state of stabilization of epigenetic and neurostructural changes responsible for the resistance of schemas to change. The cascade model developed in the present paper implies the need to modify therapeutic interventions toward a sequential approach, taking into account first the regulation of physiological processes (bottom-up), and then relational and cognitive interventions (top-down). This approach may increase the effectiveness of therapy in personality disorders, especially in cases of high resistance to treatment.

## 1 Introduction

The contemporary conceptualization of the formation of *early maladaptive schemas* (EMS) is evolving from static descriptions of cognitive structures to dynamic neurodevelopmental models (1). As J. Young points out, EMS are ubiquitous patterns consisting of memories, emotions, bodily sensations, and beliefs, shaped in the course of unmet key emotional needs during critical periods of development (2, 3). Although the origin of schemas is commonly associated with relational trauma and early childhood deprivation, the literature on the subject still shows a gap in the specification of the precise causal mechanism shaping schemas – the process by which an ephemeral childhood experience is transformed into a rigid, biological structure that affects the daily functioning of an adult (4).

This paper proposes a cascade model for understanding the etiology of schemas, attempting to integrate classical developmental theories, as well as relying on reports in the field of interpersonal neurobiology and behavioral epigenetics. At the same time, using the assumptions of modern clinical neuropsychology to explain the described phenomena, we adopt a *process-based approach*, which allows us to go beyond the traditional EMS taxonomy toward understanding the functional mechanisms explaining the rigidity of the described structures (5). We assume here that EMS, attachment style and subsequent developmental crises taken from E. Erikson’s concept, constitute a coherent causal sequence, in which biological factors and relational experiences meet in the process of crystallization of the components of personality structures, namely early maladaptive schemas.

A key concept introduced in this paper is the concept of *“biological lock*”, which we use to explain the resistance of early maladaptive patterns to change. The mechanism of “biological lock” is understood as a consequence of the coupling of the final effects of the myelination process of cortico-limbic pathways and the methylation products of genes responsible for regulating the level of stress experienced (including the gene encoding the glucocorticoid receptor - *NR3C1* and the gene encoding the protein regulating the activity of the glucocorticoid receptor - *FKBP5* (6).

## 2 Theoretical foundations of the model – the theory of J. Piaget, E. Erikson and J. Bowlby

To better understand the cascade model of the genesis of EMS, it is necessary to go beyond Young’s taxonomy itself and rely on three theoretical pillars: the mechanism of consolidation of changes (J. Piaget’s theory), time frames (E. Erikson’s theory) and biological regulator (J. Bowlby’s theory).

### 2.1 Assimilation and accommodation as a matrix of cognitive rigidity

Piaget’s theory is characterized by three key ideas: the stadial nature of cognitive development, the occurrence of cognitive patterns, and the interaction of assimilation (adaptation of new information to existing patterns) and accommodation (modification of patterns under the influence of incoming information), which enable the adaptation of the individual to the environment (7, 8). In the context of the ontogeny of early maladaptive schemas, these mechanisms take on a pathoplastic character. As Young (2) points out, schemas are characterized by extraordinary resistance to change, which in turn can be interpreted from the perspective of Piaget’s theory as the dominance of the assimilation process over the accommodation process (8, 9). Moreover, according to Leahy (10), the cognitive schemas described by Beck can be understood as developed forms of Piaget’s cognitive structures, which, formed in the early stages of development, are consolidated and manifest as cognitive distortions in adult life.

In order to further trace how the described dominance of assimilation over accommodation may translate into the development of specific early maladaptive schemas, it is necessary to break this phenomenon down into individual stages.

In the initial sensorimotor stage, the child’s experiences are primarily emotional and somatic. Due to the lack of developed symbolic structures, they are encoded in the form of non-verbal reaction patterns, which can form the basis of the so-called pre-schemas. In the preoperative stage, along with the development of language and symbolic thinking, there is a verbalization of previous experiences. Cognitive egocentrism, characteristic of this stage, promotes the internalization of negative experiences, which leads to the formation of beliefs relating to one’s own value and competence. In the stage of concrete operations, the development of logical thinking makes it possible to formulate more complex cognitive rules. However, in the conditions of negative experiences, they are overgeneralized, which results in the further consolidation of rigid beliefs. Finally, in the stage of formal operations, the ability to think abstractly and create coherent narratives about oneself and the world appears. The individual can then develop complex belief systems that serve a defensive function against previous experiences. Mechanisms such as intellectualization or rationalization favor the maintenance of maladaptive patterns through selective processing of information, which leads to their self-perpetuation (2, 11).

The key element integrating the above processes is the dominance of assimilation over accommodation in conditions of early trauma or chronic deprivation of emotional needs. The individual stops updating their patterns in response to new experiences, instead interpreting them in a way that is consistent with pre-existing beliefs. As a result, a closed cognitive-emotional system is created, which is maintained through mechanisms of cognitive distortion and selective attention and memory. (12).

The following list (**Table 1**) illustrates how the natural cognitive processes identified by Piaget, as a result of relational experiences, become the basis for the development of EMS.

**Table 1:** Stages of cognitive development according to Piaget’s theory and the formation of EMS.

Piaget’s developmental stage	Mechanism of the assimilation process	The formation of EMS
I. Sensorimotor (0–2 years)	**Affective generalization** During this period, sensory cognition dominates, and early childhood experiences are encoded on a somatic and emotional level (11). The consequence of this stage is the acquisition of the so-called immutability or permanence of the object - an ability that allows the creation of an internal cognitive representation of a given object in one’s own mind (7)	Development of pre-schema – non-verbal, emotional-somatic patterns (Young, 2003). They are based on the so-called *implicit memory* (13).
II. Preoperative (2–7 years old)	**Cognitive egocentrism** The effect of this developmental stage is interiorization and the development of internal speech, the development of concepts and symbolic play (7). A child’s thinking is characterized by egocentrism, animism, and centering and irreversibility (11). Cognitive egocentrism is characterized by the child’s inability to take a perspective other than their own, which means that the child can only perceive the world from their own point of view (7). Centration, in turn, means focusing attention on a single or narrow fragment of reality and ignoring the broader context (11). As a consequence of these two processes, the child attributes the causes of events (including negative ones) to himself, and treats the negative messages of the caregiver as representations of his own characteristics.	Schema crystallization Non-verbal experiences from the previous stage take the form of verbal beliefs, the basis of which is *explicit memory*, e.g., “I am unloved”, “Something is wrong with me” (2, 4, 13).
III. Specific operations (7–11 years old)	**Overgeneralization** The development of rule-based thinking, as a result of which individual experiences (including those tinged with negative emotionality) are generalized (11).	
IV. Formal operations > 11 years of age	**Intellectualization** The child acquires the ability to think abstractly and comprehensively, and new experiences are integrated into a coherent system of beliefs about themselves and the world (7). The mechanism of assimilation in this period takes the form of systemic integration of information, in which new experiences are not only interpreted through the prism of individual beliefs, but are incorporated into broader, coherent cognitive structures.	Systemic defense of the truthfulness of the schema through mechanisms of cognitive distortion. Complex narratives are created that support the patterns (2).

Under conditions of proper development, the accommodation process allows for constant revision and updating of internal operating models in response to new empirical data (14). However, in the case of early relational trauma or chronic deprivation of emotional needs, this mechanism is functionally blocked (9). The individual stops modifying his cognitive structures, forcing the adjustment (assimilation) of each new experience to the existing, dysfunctional pattern. As Maruszewska et al. (15) emphasize, this cognitive rigidity is not only an error in information processing, but reflects persistent neuropsychological deficits, manifested, m.in, by reduced plasticity within the prefrontal cortex and the cingulate gyrus (cf. Ramamurthy & Chen (16)).

The neurobiological basis of the stiffening of assimilation processes is confirmed in studies on developmental and stress-induced myelination of limbic pathways (17, 18). The intensive consolidation of neural connections in response to early traumatic stress (19) makes the pattern, once formed, a “neural highway” along which information moves bypassing the processes of critical verification of new stimuli. As a result, a patient with a strongly developed pattern, e.g., Defectiveness/Shame, will assimilate neutral or positive social signals as confirmation of their own inadequacy, which closes the feedback loop and prevents the accommodation of new, corrective knowledge about themselves (1).

In conclusion, Piaget’s theory provides a coherent framework for understanding the development of cognitive structures that can take on a dysfunctional character under conditions of abnormal relational experiences. The mechanisms of assimilation and accommodation, originally used for adaptation, lead to the consolidation of rigid (non-adaptive) interpretive patterns in a situation of predominance of assimilation.

### 2.2 The dynamics of developmental crises as “windows of plasticity”

In Erikson’s original theory, successive developmental stages are shaped by social influences interacting with a psychophysically maturing organism. Each subsequent stage depends on the correct course of the previous phase, and at the same time prepares and conditions the next stage (20, 21). According to the author, one of the key developmental processes is the process of integrating internal and external experience at the level of the individual’s ego, which is responsible for the coherence and individual character of development (21).

In the model we created and presented in this article, psychosocial theory is redefined and treated as a chronological (temporal) map of biological sensitive *periods*. The latter should be understood as periods in development in which the nervous system shows particular susceptibility to the influence of environmental experiences (external stimuli have an increased ability to shape the structure and function of the brain, leading to relatively permanent changes in the functioning of the individual) (6, 22). Contemporary approaches emphasize that these periods are not absolutely closed, but are phases of increased plasticity, in which experience plays a key role in the organization of neural circuits.

The biological basis of these windows consists of dynamic neurodevelopmental processes such as synaptogenesis, *synaptic pruning*, myelination, and changes in the balance between excitation and inhibition processes occurring in the central nervous system (23). Epigenetic mechanisms are also important for this phenomenon, as they enable sustained modulation of gene expression under the influence of environmental experiences, especially those of a stressful nature (24). Thus, adverse experiences such as violence, neglect or chronic deprivation of emotional needs may exert a profound influence on brain development when they occur during periods of increased biological sensitivity. In these cases, alterations occur in the structures responsible for regulating emotions and processing traumatic experiences (primarily in the region of the amygdala and hippocampus) are observed (25).

We assume that the first stages of development, described in Erikson’s theory, are related to the level of satisfaction of Young’s five basic emotional needs (**Table 2**) (2, 20, 27). In the presented theory, we focus on the first stages of the theory of psychosocial development, but it should be remembered that biological windows of sensitivity occur not only in early childhood, but also in later stages of development, including adolescence (28). From the perspective of developmental neurobiology, failure to resolve a conflict within a designated time window (e.g., in the “Trust vs Distrust” phase) results not only in a psychosocial deficit, but above all in the “biological closure” of the neural structures responsible for a given affective function (18, 29).

**Table 2:** Linking the successive phases of Erikson’s psychosocial development with Young’s theory of early non-adaptive schemas.

Age	Crisis according to Erikson	Potentially gained Strength/virtue	Early childhood needs of Young’s	EMS domain
I (0–18 months)	Trust vs Distrust The child is completely dependent on the caregivers. In order for trust to be born, the parent should love the child, anticipate his needs, accompany him. The tasks to be performed at this stage are trust in the mother or another caregiver and in one’s own causative capabilities (26).	Hope	Secure Attachment and Care	I. Disconnection and Rejection
II (1.5–3 years old)	Autonomy vs Shame At this stage, the child becomes more independent, learns to control his body more and more. New psychomotor skills give you the opportunity to make a choice. The child develops a sense of control, agency related to responsibility for independent activities. In this way, the child gains a sense of autonomy in a given area of functioning. If he is treated inappropriately, criticized, he may become ashamed (26).	Will	Autonomy and Competence	II. Weakened autonomy
III (3–6 years old)	Initiative vs Guilt The child is increasingly showing initiative in action. He is more confident, wants to explore the world, develops the ability to self-observe. At this stage, a conflict with parents may arise due to the child’s growing independence and willingness to decide for himself, which in consequence may result in disapproval of adults and arouse a sense of guilt in the child (26).	Definitely	Freedom to express your needs and emotions	IV. Targeting others
IV (6–12 years old)	Diligence vs Inferiority It is a stage of assimilating basic cultural norms and skills. Failures experienced at this stage are associated with the loss of role in the group, which causes a sense of inferiority and difficulty in identifying with the peer group. The course of development in this phase affects attitudes toward work and people later in life (26).	Competence	Realistic boundaries	III. Damaged borders / V. Over-Alertness and Inhibition

The described phenomena explain the mechanism of cascading inheritance of deficits: failure at the earliest Eriksonian stages becomes a strong predictor of the occurrence of specific EMS domains in adulthood (30). In this view, Erikson’s developmental crises are not just stages of ego formation, but critical moments for brain plasticity. Each “biological closure” of the developmental window without satisfying an adequate emotional need stiffens the maladaptive structure, creating the foundations for the previously described “biological lock” (1, 29).

### 2.3 Bonding style as a mediator of epigenetic processes

Bowlby’s (31, 32) attachment theory acts as a regulatory axis in the proposed model, connecting early relational experiences with the biological basis of personality. In the light of modern research, attachment style cannot be understood only as a behavioral pattern, but as a system of regulating physiological arousal that organizes the functioning of the stress axis and the processing of emotional information (33). As indicated by Juraś-Darowny et al. (34), attachment disorganization is an axial element of the clinical picture of personality disorders and is directly rooted in early relational experiences.

According to the approach to the relationship with the object, internal representations *of the self* and others arise on the basis of early childhood interactions and are subjective, organizing the subsequent behavior and motivation of the individual (35, 36). In this sense, attachment style can be understood as a functional manifestation of *internal working models (*IWM), which are a cognitive-affective record of relational experiences (37).

In the cascade model, a key extension of this approach is the epigenetic perspective, which indicates that relational experiences leave a permanent biological trace. Epigenetic mechanisms such as DNA methylation, miRNA regulation, and histone modifications facilitate long-term modulation of gene expression under the influence of environmental experiences (38). As Juraś-Darowny et al. (34) emphasize, traumatic experiences in early childhood can lead to permanent epigenetic changes that affect the mental and somatic functioning of the individual.

These processes may provide a biological basis for the crystallization of early maladaptive patterns, permanently impairing the body’s ability to extinguish the stress response. In this approach, the attachment style can be understood as a mediator between the environment and biological processes, through which relational experiences are transformed into permanent changes at the neurobiological level (6). As a result of the described processes, stable, difficult-to-modify patterns of functioning (i.e. early maladaptive schemas) characteristic of personality disorders are formed.

**Figure 1:**
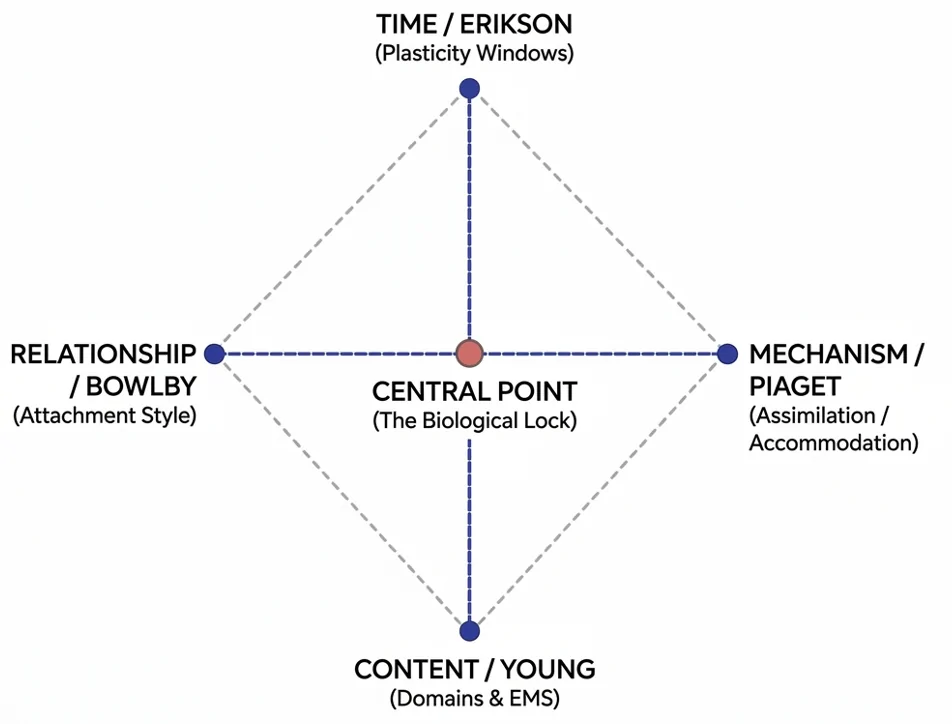
Tetrahedron of EMS ontogeny. Own study.

Summarizing the above considerations, the mutual interpenetration of the four discussed paradigms (cognitive mechanism, temporal dimension, the phenomenon of bonding and early childhood needs constituting the nucleus of EMS) is illustrated in **Figure 1**.

In this context, significant importance is attributed to the gene encoding the glucocorticoid receptor (NR3C1). Increased methylation of this gene is associated with both the experience of early childhood trauma and symptoms of borderline personality disorder (39). Expression of the NR3C1 gene affects, among other things, the regulation of *hypothalamic-pituitary-adrenal axis* (HPA) and hormonal stress response, and its epigenetic changes are observed in the case of stress experienced in the early stages of life, among other things, as a result of exposure to abuse or neglect by the caregiver (40, 41). Also in the healthy adult group, elevated NR3C1 methylation has been associated with a tendency to avoid proximity, which in turn indicates the potential importance of HPA axis action and the regulatory role of social stress in shaping attachment style and epigenetic modifications as its biomarkers (42).

## 3 Biological basis of the cascade model and integration of its individual elements

**Figure 2:**
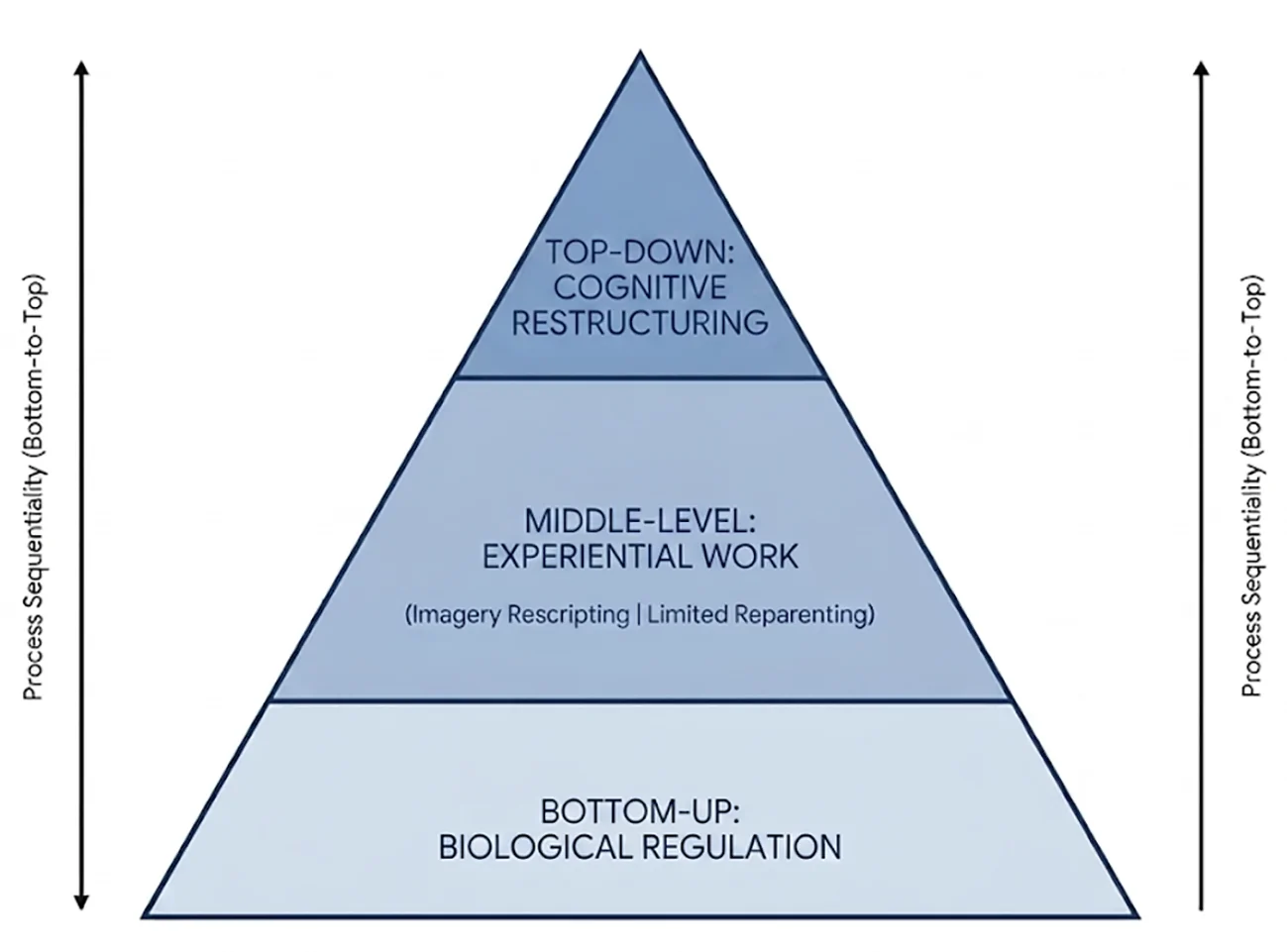
Causal model of EMS ontogeny (synthesis). Own study.

In the proposed cascade model, the biological basis for the development of early maladaptive schemas can be understood as the process of sequential “coding” of relational experiences in neurobiological and cognitive structures. In the spirit of dynamical systems theory (43, 44), it is assumed that disorders occurring in the early stages of development are not isolated, but initiate a cascade of changes that determine the availability of resources necessary for adaptive resolution of subsequent developmental crises. Thus, EMS ontogeny is a cumulative process in which environmental experiences undergo biological consolidation, e.g., through epigenetic mechanisms (45). A graphical presentation of the model is shown in **Figure 2**.

The first link in the developmental cascade falls on the infant period, corresponding to the “Trust vs Distrust” phase, in which the basic pattern of the relationship with the caregiver and the sense of security are formed (31). From a neurobiological perspective, the asynchronous development of brain structures is crucial during this period - the hippocampus, responsible for declarative memory and narrative integration of experiences, has not yet reached full functional maturity (46). As a consequence, relational experiences of a stressful nature are not encoded in the form of conscious cognitive representations, but are recorded at the somatic-affective level, with the significant participation of the amygdala, which plays a central role in the processing of threat (6, 19).

At this stage, epigenetic processes are of particular importance. Chronic stress resulting from the lack of stable care or the unpredictability of the care environment leads to changes in the expression of genes that regulate the stress response. In particular, it has been shown that there is an increased methylation of the NR3C1 gene, encoding the glucocorticoid receptor, which results in a disorder of the inhibition mechanisms of the hypothalamic-pituitary-adrenal axis (6, 47). As a result, the stress response is prolonged and perpetuated, even in the absence of a real threat.

The consequence of these processes is the formation of a nervous system with increased sensitivity to potentially threatening stimuli. On a psychological level, this is the basis for the development of patterns from the domain of Disconnection and Rejection, such as Abandonment or Distrust/Harm. In this approach, these schemas are not only cognitive structures, but reflect deep-rooted patterns of biological reactivity that affect the functioning of the individual even under conditions of objective safety (1, 29). A deficit in primary security also limits the ability to explore the environment, which is a significant risk factor for further cognitive and emotional development.

At the next stage, corresponding to the “Autonomy vs Shame” phase (27), a sense of agency and control over one’s own actions develops. This process is related to the functioning of the dopaminergic system, responsible for motivation and reward processing, and to the activity of the insular cortex, which integrates interoceptive and emotional signals. In conditions of adequate support from caregivers, the child’s natural tendency to explore the environment is conducive to shaping autonomy. However, in a situation of excessive control, criticism or emotional deprivation, autonomous activity is associated with negative affect, in particular shame (31, 48).

At the neurobiological level, this process may be associated with dysregulation of the so-called *seeking system*, described by Panksepp (49), and changes in the functioning of the striatum, which plays a key role in initiating goal-oriented behaviors (34). At the same time, the insular cortex may show increased reactivity to social experiences, which promotes the intensification of shame affect and its somatization (29, 50). As a result, patterns from the domain of Weakened Autonomy develop, including Dependence/Incompetence and Failure (2). The lack of adequate experiences that strengthen agency leads to the consolidation of beliefs about one’s own ineffectiveness and a tendency to avoid challenges.

With the maturation of cortical structures and the transition to the next stages of psychosocial development (“Initiative vs Guilt” and “Diligence vs Inferiority”; 20, 27), anxiety undergoes further transformation and begins to be integrated into cognitive processes. At the preschool stage, the *anterior cingulate cortex* (ACC) plays an important role, involved in monitoring conflicts and assessing errors, especially in the social context (29, 50). In conditions of chronic stress, its hyperactivity can lead to excessive concentration on signals of disapproval and to an increase in self-criticism.

As a consequence, the child may develop adaptive strategies of giving up their own needs in favor of maintaining a relationship with their caregiver, which is reflected in patterns such as Self-Sacrifice or Submission (48). At the school stage, myelination processes occurring in the central nervous system (CNS) lead to an increase in the efficiency of nerve impulse conduction, but at the same time they promote the consolidation of frequently activated neural patterns (17). As a result, cognitive patterns such as Ruthless Standards or Emotional Inhibition are becoming more and more stable and automatic.

According to Piaget’s (8) concept of cognitive development, at this stage, developing operational thinking enables the child to create coherent narratives about his or her own experiences. This process finds its neurobiological basis in the maturing prefrontal cortex (PFC), which becomes the main center for the integration of raw affective data into the emerging belief structures. In conditions of a predominance of negative experiences, this leads to the emergence of cognitive justifications for the suffering experienced, which can take the form of intellectualization. As Mącik (9) points out, schemas cease to serve only as an adaptive reaction and become an integral element of the personality structure. Established cognitive-emotional patterns show significant resistance to change, which can be interpreted as the effect of stabilization of neurobiological processes.

The final stage of the described cascade is a state we refer to as a “ *biological lock*”, understood as a relatively permanent configuration of epigenetic and neurostructural changes. It includes, m.in, fixed methylation of genes related to stress regulation (e.g., NR3C1, FKBP5) and changes in the functioning of the limbic system, including increased amygdala reactivity (6, 17). In this state, the primary experiences of anxiety and shame become integrated into secondary cognitive structures, making them significantly more difficult to modify with strategies based solely on cognitive processing.

From a neurobiological perspective, attempts to change patterns can be interpreted by subcortical structures as a threat to the integrity of the regulatory system, resulting in activation of defense mechanisms and resistance to change (19, 50). As a result, the body prefers to maintain established, albeit dysfunctional, patterns of functioning that provide relative predictability and a sense of control.

**Table 3** below shows an integrated matrix of factors influencing EMS crystallization.

**Table 3:** Factors influencing the crystallization of early maladaptive schemas (6, 19, 47).

Cascade level	Development crisis (Erikson)	Cognitive mechanism (Piaget)	Key Need (Young)	Biological agents	Resulting EMS domain
I (0–18 months)	Trust vs Distrust	affective generalization	Secure attachment and care	amygdala, hippocampus, HPA axis (51), NR3C1 gene (6), cortisol (52)	Disconnection and Rejection
II (1.5–3 years old)	Autonomy vs Shame	cognitive egocentrism	Autonomy and competence	striatum (reward system), insular cortex (53), FKBP5 gene (54), dopamine (55)	Weakened autonomy
III (3–6 years old)	Initiative vs Guilt	overgeneralization	Freedom to express needs	anterior cingulate gyrus (ACC) (56), OXTR gene (57)	Targeting others
IV (6–12 years old)	Diligence vs Inferiority	Intellectualization	Realistic boundaries and self-control	prefrontal cortex (PFC) (58), BDNF (59), myelination (60)	Damaged Boundaries/Over-vigilance

To sum up, the development of EMS is cascading. Each of the four levels presented acts as a filter that gradually “condenses” the original emotional experiences, transforming them into increasingly rigid cognitive-behavioral mechanisms. The end result of this process is a fixed state of “biological lock”.

At this point, it should be emphasized that the development of early non-adaptive schemas does not begin only after birth but may have its determinants already in the prenatal period (61). A growing body of evidence suggests that the mother’s psychophysiological state, including stress levels and emotional regulation patterns, can affect the developing fetal nervous system through neuroendocrine mechanisms (62). Chronic exposure to elevated levels of glucocorticoids, especially cortisol, may contribute to fetal programming, a process of developmental plasticity through which the fetus adapts to intrauterine environmental conditions (63), in which the basic parameters of the stress-response system are shaped. These mechanisms are associated with permanent epigenetic changes, particularly within genes such as *NR3C1* (38, 64), although it is now recognized that the effects of prenatal and early-life stress involve multiple genes, interacting neural systems, and biological pathways beyond the HPA axis (65).

As a result, the newborn may exhibit constitutionally conditioned heightened sensitivity to emotional stimuli and a lowered threshold of anxiety response. In the literature, this phenomenon is described as early biological *sensitivity to context*, which is the basis for the later development of emotional-behavioral patterns (66). As indicated by Oberlander et al. (64) and Talarowska (52), changes in the functioning of the HPA axis can lead to persistent stress hyperreactivity from the early stages of life. Studies on the epigenetics of affective disorders have also shown that early stress experiences are associated with changes in gene expression related to stress regulation and neuroplasticity, which increases susceptibility to developing mental disorders later in life (52). As a consequence, the infant’s body may react to relatively minor environmental disturbances in a manner characteristic of a reaction to a threat. In the context of the cascade model, the prenatal phase can therefore be understood as a baseline level, determining the individual sensitivity of the body to environmental experiences and the rate of consolidation of successive layers of dysfunctional regulation patterns (6, 38).

Another particular developmental stage in the described cascade is the period of adolescence, characterized by both increased neurobiological plasticity and a tendency to stabilize previously formed patterns of functioning. During this time, intensive reorganization processes take place within the brain, including, m.in, the selective elimination of neuronal connections and the maturation of the prefrontal cortex, responsible for cognitive regulation and behavior control (67, 68). This period is associated with dynamic changes in the functioning of the limbic system and its connections with the prefrontal cortex, which increases emotional reactivity and susceptibility to environmental influences (52, 69). These processes enable the integration of earlier, mainly somatic-affective records of experience with the developing personality structure (schemas). In this sense, adolescence can serve as a period of increased susceptibility to modify previous deficits, especially in the context of new relational experiences such as peer bonds or mentoring relationships. At the same time, however, it is the moment when previously established patterns can be further stabilized. In the case of the dominance of negative experiences, there is a reorganization of patterns at the cognitive level – previous, non-verbal emotional representations are transformed into a relatively coherent system of beliefs about oneself and the world. As Mącik (9) points out, it is during adolescence that previously dispersed patterns of reactions are integrated and take the form of a stable identity narrative. From a neurobiological perspective, this process runs parallel to the consolidation of specific patterns of neuronal activity and epigenetic changes, which promotes the stabilization of dysfunctional cognitive-emotional patterns (52).

## 4 Proposal of therapeutic interventions

**Figure 3:**
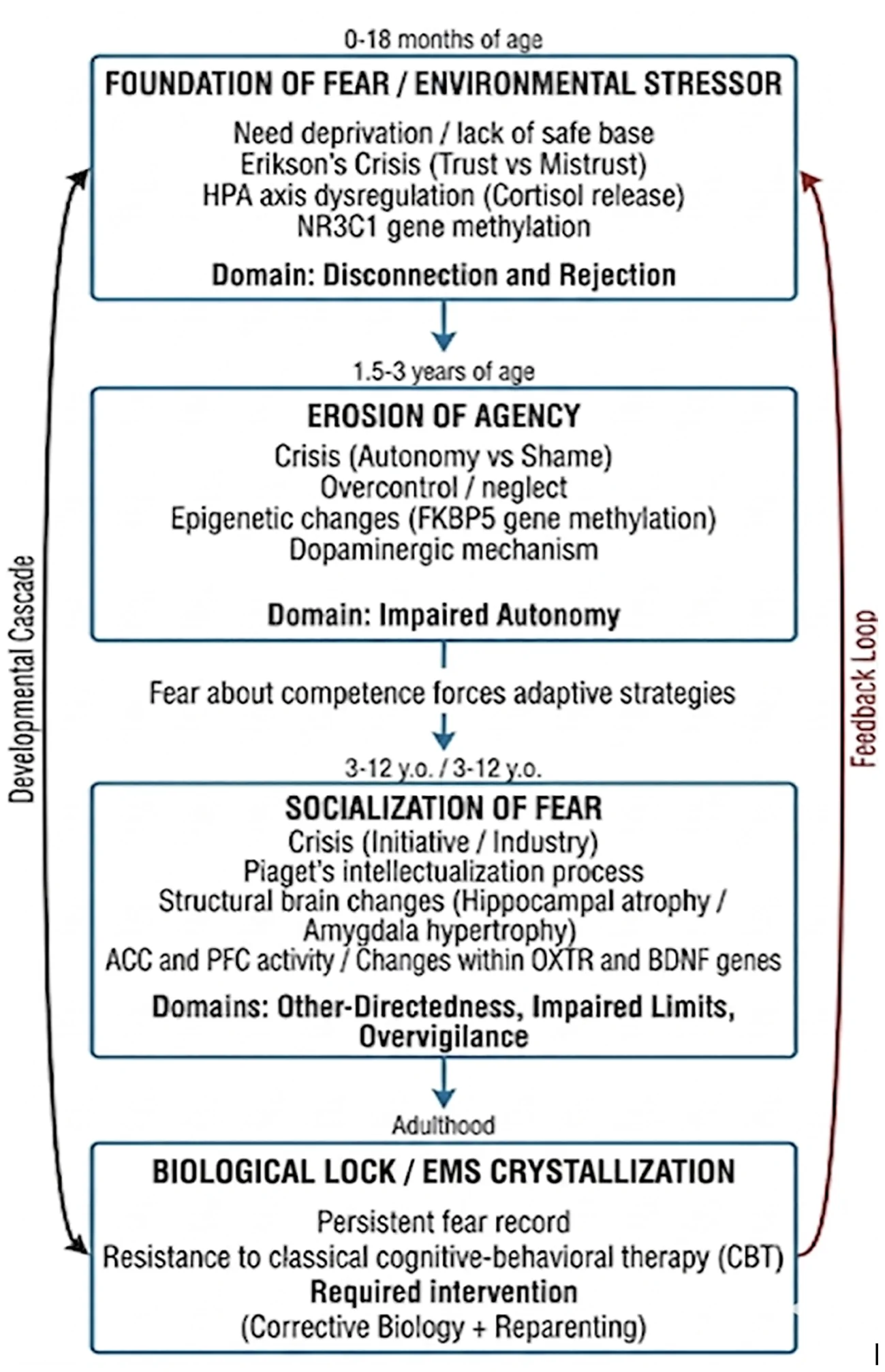
Pyramid of cascade intervention. Own study.

The conceptualization of early maladaptive patterns in terms of “biological lock” implies the need to revise existing strategies of therapeutic interventions. Assuming that schemas are not merely distorted cognitive structures, but reflect established patterns of neurobiological activity, requires an adequate adaptation of clinical intervention methods. In the proposed model (**Figure 3**), it is assumed that the selection of therapeutic techniques should take into account the developmental stage and the level of organization of the central nervous system at which a given schema was formed.

The earliest patterns, corresponding to levels I and II (infancy and post-infancy), are formed primarily within the subcortical structures, with limited participation of language and reflective systems. As a consequence, cognitive interventions based on top-down processing may show limited effectiveness in the early stages of therapy, especially in patients with profound personality disorders. The cascade model therefore indicates the need to use an orderly sequence of therapeutic interventions, in which approaches aimed at regulating somatic and autonomic processes (*bottom-up* interventions) are given priority.

These interactions focus on modulating the activity of the autonomic nervous system and integrating somatic-affective records related to stress experiences. Their goal is to reduce the excessive reactivity of limbic structures, in particular the amygdala, and restore the ability to adaptively process emotional arousal, described as functioning in the so-called tolerance window (19, 70). As Talarowska (50) emphasizes, stabilization at the physiological level is a necessary condition for the effective involvement of higher cortical structures, including the prefrontal cortex, responsible for cognitive control and emotion regulation.

Only after achieving a relative balance in terms of autonomic regulation does it become possible to introduce an intervention of a relational nature. In this context, *the technique of limited reparenting* can be understood as a mechanism modulating the functioning of neurobiological systems related to attachment and stress. The experience of the therapeutic relationship as a safe base (31) is associated with the activation of neuropeptide systems, including oxytocin, which plays an important role in the regulation of the stress response and in affiliation processes.

This process may contribute to the reduction of the activity of the hypothalamic-pituitary-adrenal axis and promote an increase in neuronal plasticity, which in the long term creates conditions for the modification of established regulatory patterns (6, 71). In this approach, the therapeutic relationship acts as a corrective factor at the biological level, enabling a gradual reorganization of previously established stress reactions.

Advanced cognitive and narrative interventions, characteristic of higher levels of organization (III and IV), involving work on identity and life history, achieve optimal effectiveness only when they are embedded on a previously stabilized neurobiological substrate. As Siegel (29) and Reubsaet (1) point out, the integration of emotional and cognitive experiences requires the ability to regulate affect to be restored beforehand. Such a hierarchy of therapeutic interventions allows for the gradual “unlocking” of established patterns of reactivity and the introduction of more permanent changes in the structure of personality functioning.

Therefore, the fundamental stage of the therapeutic process, corresponding to the level of *bottom-up* interactions, is the restoration of adequate physiological regulation of the autonomic nervous system and the hypothalamic-pituitary-adrenal axis. This is the level of the next stage of experiential work, in which the therapeutic relationship plays a key role as a corrective factor, operationalized, e.g., within the concept of limited re-parenting. As indicated by research on the biological basis of the stress response and attachment (6, 71), achieving relative stabilization at the level of subcortical processes is a necessary condition for effective involvement of cognitive systems. Only on such a well-established basis is it possible to implement cognitive interventions, including the restructuring of beliefs and work on narrative identity (*top-down level*).

A sequential approach to the therapeutic process, in accordance with the cascade model, reduces the risk of premature use of verbal techniques against established neurobiological patterns. As Reubsaet (1) and Mącik (9) point out, skipping the stage of physiological regulation is an important factor promoting treatment resistance in the case of profound personality disorders.

## 5 Summary

The synthesis of the theoretical frameworks proposed by Piaget, Erikson, Bowlby, and Young has enabled the development of an original cascade model explaining the ontogeny of early maladaptive schemas (EMS). The present analysis suggests that EMS should not be understood merely as isolated cognitive constructs but rather as the result of a sequential and integrated process through which the organism adapts to chronic deprivation of core emotional needs (2, 31, 72).

According to the proposed model, schema pathology develops progressively across successive developmental stages. In clinical practice, this process can be observed in patients for whom each successive stage of the cascade of fear and shame has been shaped by biological and relational failures occurring during the preceding developmental period (27, 29).

The concept of the biological lock, introduced in the present paper, provides a neurobiological explanation for the persistence of early maladaptive schemas and their resistance to therapeutic change. By linking the stability of personality structures to processes such as myelination of limbic pathways (17) and epigenetic modifications, including methylation of the NR3C1 and FKBP5 genes (6, 47), the proposed model offers a biological rationale for the limitations of interventions based exclusively on cognitive (top-down) techniques. In clinical practice, this phenomenon is reflected in patients who are able to understand their difficulties on an intellectual level but continue to respond to stressful situations with panic, dissociation, or emotional shutdown.

The proposed cascade model suggests that psychotherapy for severe personality disorders may benefit from a sequential therapeutic approach that takes into account both neurobiological and psychological mechanisms underlying the development and maintenance of EMS. Rather than relying exclusively on cognitive restructuring, therapeutic interventions should first focus on restoring physiological regulation and reducing maladaptive stress reactivity, thereby creating conditions that facilitate subsequent relational and cognitive change. Such an approach may enable the gradual modification of dysfunctional patterns established during sensitive developmental periods (18, 48).

Finally, the integration of bottom-up experiential interventions, corrective relational experiences, including limited reparenting (1), and contemporary neuropsychopharmacological approaches aimed at reopening windows of neuroplasticity (73) may provide new opportunities for improving treatment outcomes in individuals with severe personality disorders. Although the proposed model requires empirical verification, it offers a comprehensive theoretical framework that may contribute to a better understanding of EMS ontogeny and inspire future research and clinical practice (52, 74).

## 6 Model limitations

The presented cascade model, despite its coherence at the neurobiological level, has significant limitations that require further empirical verification. It should be emphasized that the process referred to as “biological lock” does not proceed with the same intensity in all individuals. Clinical data indicate significant heterogeneity in the course of development, even in groups of people with comparable levels of exposure to early childhood traumatic experiences. This variation suggests the existence of moderating factors that can interrupt or modify the course of the pathological cascade.

In this context, special importance is attributed to later relational experiences. Research on epigenetics and neuroplasticity indicates that the presence of a secure attachment figure in later stages of life may act as a compensatory factor, influencing the regulation of stress systems (47, 71). It is indicated that qualitative interpersonal relationships may modify the expression of genes associated with the stress response, including genes that regulate the functioning of the HPA axis, potentially limiting the perpetuation of dysfunctional regulatory patterns.

An important factor differentiating the course of the described cascade is also individual variability in the field of neuroplasticity. The rate of stabilization of neural patterns and the extent of susceptibility to their modification show significant individual variability (17). In some people, increased plasticity of the nervous system is observed for a longer time, which increases the possibility of integrating corrective experiences, including therapeutic effects. As a consequence, even established patterns of functioning can be modified relatively quickly.

Another important moderator is temperament, understood as a relatively constant set of biologically determined traits that regulate emotional and behavioral responses. Research in the field of behavioral genetics (75) and the assumptions of the Regulatory Theory of Temperament (76) indicate that such traits as emotional reactivity or the level of excitability can be biologically determined to a large extent. Consequently, individual temperamental predispositions can modulate the perception and processing of relational experiences. In practice, this means that in some people, increased sensitivity of the nervous system can lead to an intensification of responses to environmental stimuli, even in conditions of relatively adequate care. Thus, the initiation of processes leading to the development of dysfunctional patterns can occur not only in response to overt relational deficits, but also as a result of the interaction between the environment and innate temperamental characteristics.
